# WO_3_ Thin-Film Optical Gas Sensors Based on Gasochromic Effect towards Low Hydrogen Concentrations

**DOI:** 10.3390/ma16103831

**Published:** 2023-05-19

**Authors:** Michał Mazur, Paulina Kapuścik, Wiktoria Weichbrodt, Jarosław Domaradzki, Piotr Mazur, Małgorzata Kot, Jan Ingo Flege

**Affiliations:** 1 Faculty of Electronics, Photonics and Microsystems, Wroclaw University of Science and Technology, Janiszewskiego 11/17, 50-372 Wroclaw, Poland; paulina.kapuscik@pwr.edu.pl (P.K.); wiktoria.weichbrodt@pwr.edu.pl (W.W.); jaroslaw.domaradzki@pwr.edu.pl (J.D.); 2 Institute of Experimental Physics, University of Wroclaw, Max Born 9, 50-204 Wroclaw, Poland; pioma@ifd.uni.wroc.pl; 3 Applied Physics and Semiconductor Spectroscopy, Brandenburg University of Technology Cottbus-Senftenberg, Konrad-Zuse-Strasse 1, D-03046 Cottbus, Germany; sowinska.gosia@gmail.com (M.K.); flege@b-tu.de (J.I.F.)

**Keywords:** WO_3_, gas impulse magnetron sputtering, thin film, gasochromic properties, optical properties, annealing, optical hydrogen gas sensor

## Abstract

Hydrogen gas sensors have recently attracted increased interest due to the explosive nature of H_2_ and its strategic importance in the sustainable global energy system. In this paper, the tungsten oxide thin films deposited by innovative gas impulse magnetron sputtering have been investigated in terms of their response to H_2_. It was found that the most favourable annealing temperature in terms of sensor response value, as well as response and recovery times, was achieved at 673 K. This annealing process caused a change in the WO_3_ cross-section morphology from a featureless and homogenous form to a rather columnar one, but still maintaining the same surface homogeneity. In addition to that, the full-phase transition from an amorphous to nanocrystalline form occurred with a crystallite size of 23 nm. It was found that the sensor response to only 25 ppm of H_2_ was equal to 6.3, which is one of the best results presented in the literature so far of WO_3_ optical gas sensors based on a gasochromic effect. Moreover, the results of the gasochromic effect were correlated with the changes in the extinction coefficient and the concentration of the free charge carriers, which is also a novel approach to the understanding of the gasochromic phenomenon.

## 1. Introduction

Tungsten trioxide (WO_3_) is a wide-bandgap (~2.6–3.3 eV) semiconductor. Depending on the deposition temperature, WO_3_ can occur in various crystal structures, including the monoclinic phase (up to 330 °C), orthorhombic phase (330–740 °C), and tetragonal phase (above 740 °C) [1,2]. Its advantages include high chemical stability and low price [3,4]. Furthermore, WO_3_ has chromogenic properties, that is, the ability to switch between pale yellow and dark blue colour in response to various stimuli, including temperature, light irradiation, electric field, and exposure to hydrogen [1,3,4]. Due to its high colouring efficiency and fast switching, it can be used in gas sensors, antidazzling mirrors, and smart window applications [1,2,3,4].

Usually, gasochromic WO_3_ thin film gas sensors require the use of noble metal nanoparticles as a catalyst, allowing the absorption and dissociation of H_2_ molecules. Various mechanisms have been proposed to explain the subsequent colouration process. In one of the proposed colouration mechanisms (sometimes called the ‘generation of oxygen vacancies’ model), the H^+^ ions interact with WO_3_, leading to the reduction of W^6+^ on the surface of the material and the formation of oxygen vacancies. The created colouration centres diffuse into the bulk of the material, while the water (by-product of the redox reaction) desorbs from the surface. In the bleaching process, the interaction with atmospheric oxygen leads to the oxidation of W^5+^ species back to W^6+^ and the increase in the transparency of the material [1,3].

Thin films of WO_3_ for application in gasochromic sensing can be prepared using a variety of methods, including sol-gel [5], electron beam evaporation [6], thermal evaporation [7], pulsed laser deposition [8,9], and magnetron sputtering [10]. To enhance the sensing response, an overlayer of Pd [6,8,10,11], Pt [7,8,12,13], or Au [14] nanoparticles can be used. These noble metals can also be used as dopants during WO_3_ film preparation [15]. In the work of Nishizawa et al. [15], the gas detection performance of Pt- and Pd-doped WO_3_ thin films was compared. The colouring rate after exposure to 4% of H_2_ was found to be faster for the Pt-WO_3_ films. The response characteristics of both types of coatings were highly repeatable. The sensing performance of WO_3_ thin films with Pt and Pd catalysts was also compared by Garavand et al. [8]. The colouration efficiency was higher for Pt/WO_3_ coatings at operating temperatures between 90 °C and 200 °C, while the Pd/WO_3_ coatings exhibited a high sensing response below this temperature range. In the work of Lee et al. [10], the Pd catalyst was deposited by e-beam evaporation on amorphous WO_3_ thin films prepared by RF magnetron sputtering. It was found that the coatings exhibited a decrease in the transmittance level of 50% in response to 1% H_2_. The sensing response was reversible for more than 1000 cycles of 30 s colouring and 30 s bleaching, but after more than 1500 cycles, the recovery of the transmittance level in the bleached state was diminished. Cho et al. [6] manufactured Pd/WO_3_ thin film sensors with high selectivity for hydrogen and a linear dependency between sensor response and gas concentration. The recovery time of the sensors was equal to 16 s.

In the present paper, an innovative gas impulse magnetron sputtering method was used for the deposition of the WO_3_ thin films and their gasochromic response towards hydrogen was investigated. As a catalyst, a 5 nm thick Pd overlayer on top of WO_3_ was used. Interestingly, this coating, after post-process annealing at 673 K, exhibited a high sensing response of 6.3 at an H_2_ concentration level as low as 25 ppm. To the best of our knowledge, it is the lowest value published to date in the case of the WO_3_ gasochromic reaction to hydrogen. We suppose that the origin of such a high sensitivity at a very low H_2_ concentration may originate from the intrinsic properties of the WO_3_ film prepared with the gas impulse magnetron sputtering [4].

## 2. Materials and Methods

WO_3_ thin films were deposited using the magnetron sputtering process with a gas impulse, whose detailed description was presented in [4]. The sputtering process lasted for 45 min, which allowed the deposition of a tungsten oxide thin film with a thickness of approximately 420 nm. During sputtering, the average pressure in the chamber was ca. 5 × 10^−3^ mbar. The gas pulses contained an Ar:O_2_ mixture with a 7:1 ratio and were injected directly on the target surface with a frequency of 10 Hz resulting in a repeatable initiation of the glow discharge. In addition, the power to the magnetron was synchronised with the gas pulses, and each power cycle was 30 ms, followed by a pause of 70 ms. High-purity metallic tungsten target was used for sputtering, and the thin tungsten oxide films were deposited on SiO_2_ (for optical measurements) and Si (for SEM measurements) substrates placed at a distance of 160 mm from the target. Furthermore, for gasochromic measurements, a thin 5 nm Pd layer, acting as a catalyst, was deposited on the surfaces of the WO_3_. After deposition, WO_3_ thin films covered with a top Pd layer were annealed in air at atmospheric pressure for 4 h at 473 K, 573 K, and 673 K in a quartz tubular furnace.

The diffraction patterns of the as-deposited and annealed thin films were determined using grazing incidence X-ray diffraction (GIXRD) with an incidence angle of CuKα radiation (wavelength of 1.5406 Å) of 3° relative to the sample surface. Empyrean PANalytical X-ray diffractometer containing a PIXel3D detector was used, and measurements were carried out with a step of 0.05° and time per step of 5 s in the 2θ range of 20 to 70°. The measurement results were analysed using MDI Jade 5.0 software, while the average crystallite size for nanocrystalline thin films was calculated using Scherrer’s equation [16].

Furthermore, the change in surface and cross-section morphology with annealing temperature was investigated by a high-resolution field-emission scanning electron microscope FE-SEM (FEI Helios Xe-PFIB).

Measurements of the transmission coefficient were carried out in the wavelength range of 300–1000 nm with the use of an OceanOptics QE65000 spectrophotometer and a DH-BAL 2000 deuterium-halogen light source. Based on those results, the fundamental absorption edge (λ_cut-off_) and the optical band gap energy (E_g_^opt^) were calculated employing Tauc plots for the latter [17]. The transmission spectra of the WO_3_ thin films covered with the Pd catalyst were measured with and without the presence of diluted hydrogen in argon using a cryostat and the experimental setup shown in [18].

Gasochromic measurements were performed at 423 K using hydrogen as an activating agent with concentrations ranging from 200 ppm to 1000 ppm, while for the thin film with the best sensor response (SR), the concentration was in the range of 25 ppm to 1000 ppm. The recovery of the thin films was ensured with synthetic air provided by an Atlas Copco GX3 FF compressor with an integrated refrigerant air drier that provided a humidity level of <10% and an activated carbon adsorbing filter (Donaldson AK series). Each measurement cycle lasted one hour, during which hydrogen was introduced into the chamber for 30 min, and then the samples were recovered using a synthetic air flow for another 30 min. Gas flow was controlled using MKS mass flow controllers. Furthermore, the extinction coefficient (k) spectral characteristics of the thin films before and during the gasochromic process were determined by the reverse engineering method using SCOUT software [19] based on the measured transmission spectra before and during hydrogen exposure. The best match of the theoretically generated with experimentally measured characteristics was obtained using Lorentz, Tauc, and Drude oscillators.

In addition, the surface oxidation state of the WO_3_ thin film annealed at 673 K, and showing the best SR, was measured using X-ray photoelectron spectroscopy (XPS). For this investigation, the Specs Phoibos 100 MCD-5 hemispherical analyser with a nonmonochromatic Specs XR-50 X-ray source with Mg Kα (1253.6 eV) radiation was used. Detailed scans were focused on photoelectron peaks related to tungsten (W4f) and oxygen (O1s). XPS investigations were carried out before and after in-situ exposure to the hydrogen at 423 K, which are referred to in the article as WO_3_ before hydrogenation and WO_3_ after hydrogenation, respectively. Before exposure to the hydrogen gas, the base pressure in the XPS chamber was ca. 10^−10^ mbar. Then, the WO_3_ thin film was heated up to 423 K, and hydrogen was introduced into the chamber, increasing the base pressure to 10^−6^ mbar, which lasted for 30 min. Afterwards, the chamber was evacuated to the initial pressure, and the sample was cooled down to room temperature. After these conditions were reached, the XPS measurement of the same regions as before was performed.

## 3. Results and Discussion

**Figure 1:**
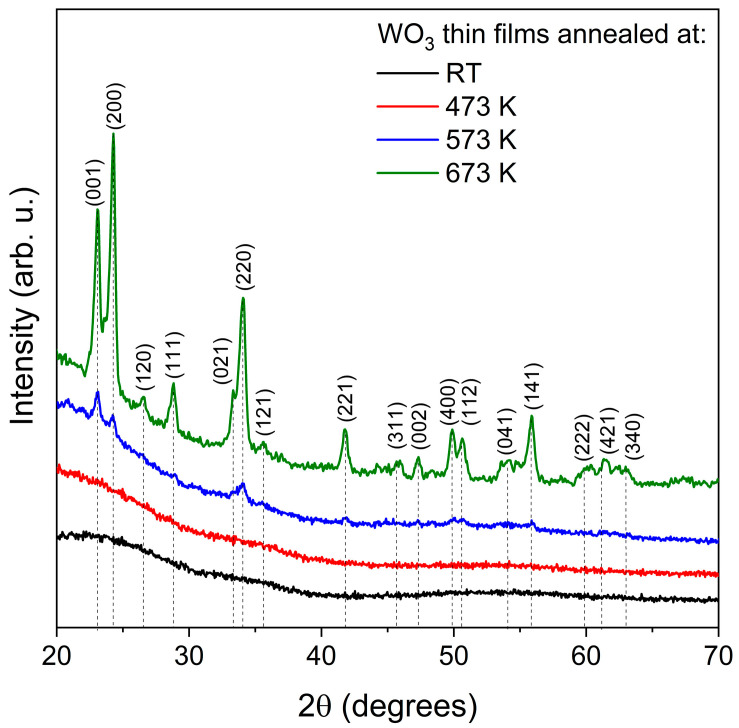
XRD patterns of WO_3_ thin films annealed at various temperatures.

Grazing incidence X-ray diffraction measurements (Figure 1), which are destined for thin film samples, showed that the as-deposited and annealed at 473 K WO_3_ thin films were amorphous. Very broad peaks seen in the 2θ range of 20–25° resulted from the amorphous fused silica substrates on which the films were deposited. It is worth noting that the thin films were deposited without additional heating or biasing of the substrates. The amorphous nature of magnetron-sputtered WO_3_ films deposited at room temperature has also been reported by others [20]. Next, post-process annealing above 573 K caused a start of the phase transition from amorphous to the nanocrystalline orthorhombic-WO_3_. This thin film still seems to be composed of a major amorphous phase since the peaks related to crystalline WO_3_ are rather small intensities. The most intense peak is assigned to the (001) crystal plane; therefore, the crystallite size was calculated taking this into account, and it was equal to 21.1 nm. Further annealing at 673 K resulted in a significant increase in the peaks’ intensities, which means further crystallisation and a decrease in the volume of the amorphous of WO_3_ thin films. The most intense peak was assigned to the (200) crystal plane, and the crystallite size for this plane was 23.0 nm.

**Figure 2:**
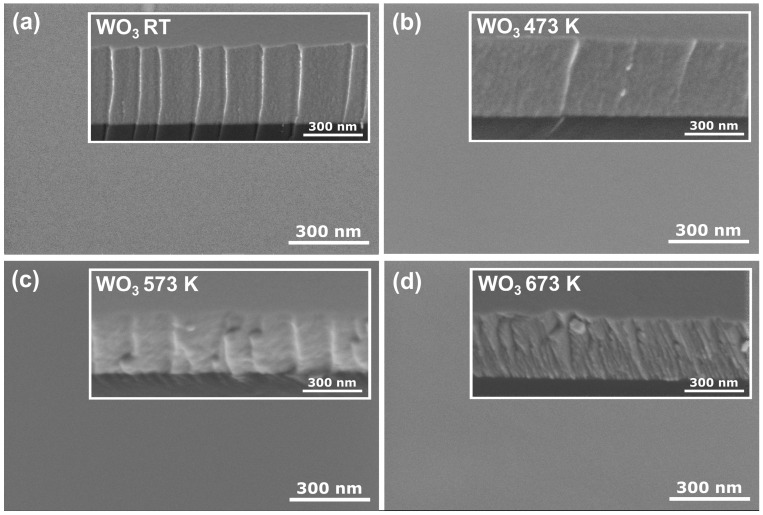
Surface and cross-section (inset) SEM images of WO_3_ thin films annealed at (**a**) RT, (**b**) 473 K, (**c**) 573 K, and (**d**) 673 K.

Figure 2 shows the surface and cross-section morphology of the WO_3_ coatings annealed at various temperatures obtained by FE SEM. All coatings had a very smooth surface with no visible grains or cracks. Similar observations were made by Limwichean et al. [21] and Besozzi et al. [22], who deposited WO_3_ films at low pressure, simultaneously favouring ad-atom growth of the thin films, resulting in their compact and uniform surface and cross-section morphology. The cross-section revealed a uniform, dense, void-free, and featureless structure for as-deposited and annealed at 473 K thin films. Although the thin film annealed at 573 K started to crystallise to orthorhombic-WO_3_ structure, the intensities of the peaks in the XRD pattern were rather small, pointing out the vast amorphous component. Furthermore, it seems that starting the transition from the amorphous phase to the nanocrystalline phase did not influence much the surface and cross-section morphology of the investigated sample. However, after annealing at 673 K, the cross-section morphology changed to a fine fibrous structure, which could result from the transition from an amorphous to a nanocrystalline structure.

**Figure 3:**
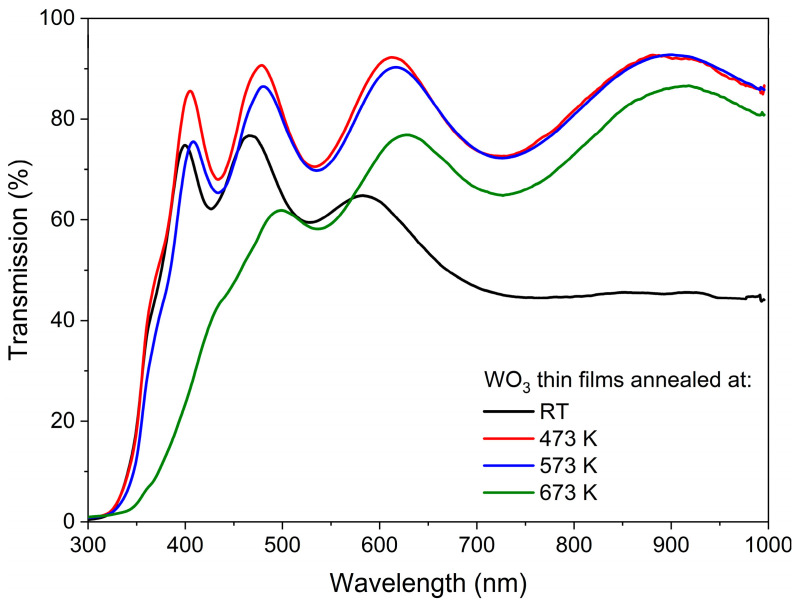
Transmission spectra of WO_3_ thin films annealed at various temperatures.

The transmission characteristics of the as-deposited and annealed WO_3_ thin films are shown in Figure 3. In each case, the interferences are seen as a common phenomenon for thin films with thickness exceeding 100 nm. WO_3_ directly after deposition had a light, pale blue tint, which can be explained by the rather high Ar:O_2_ ratio during deposition, and its transmission gradually decreased with wavelengths greater than 450 nm. Such a blue colour of sub-stoichiometric tungsten oxide thin films has also been described by others [23,24,25], and it was caused by the absorption of free carriers resulting from the presence of oxygen vacancies and increased carrier density. Annealing in the air at 473 K resulted in an increase in transmission in the whole measured wavelength range, which was presumably caused by the oxidation of the WO_3_ films and the reduction of the number of oxygen vacancies. Moreover, annealing of WO_3_ at 573 K caused only a slight decrease in transmission at lower wavelengths as compared to annealing at 473 K, which could be related to the starting point of the amorphous to the nanocrystalline phase transition. Further annealing at 673 K resulted in a more significant decrease in transmission in the measured wavelength range, as the samples were highly crystalline according to the XRD analysis.

**Figure 4:**
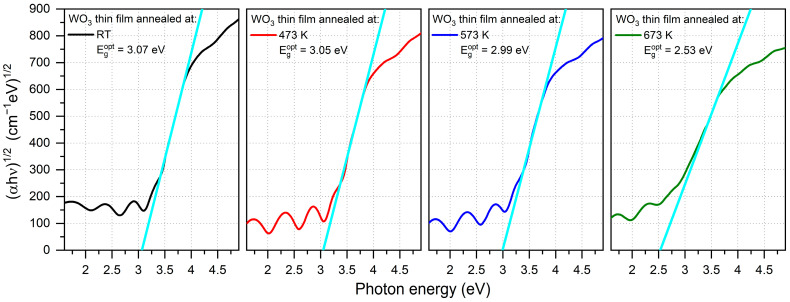
Tauc plots of WO_3_ thin films annealed at various temperatures with calculated optical energy band gaps. The light blue line is an extrapolation of the Tauc plot used to calculate the optical band gap energy.

The optical band gap energy (E_g_^opt^) was calculated using Tauc plots for indirect transitions and was in the range of 2.53 eV to 3.07 eV (Figure 4), respectively, with the decreasing post-process annealing. Values of E_g_^opt^ calculated for the amorphous thin films were much higher than for a crystalline WO_3_, which is a common phenomenon also observed by others [26,27]. A significant decrease in the optical band gap energy of the thin film annealed at 673 K may be a result of the strong enhancement of crystallinity [20,28].

**Figure 5:**
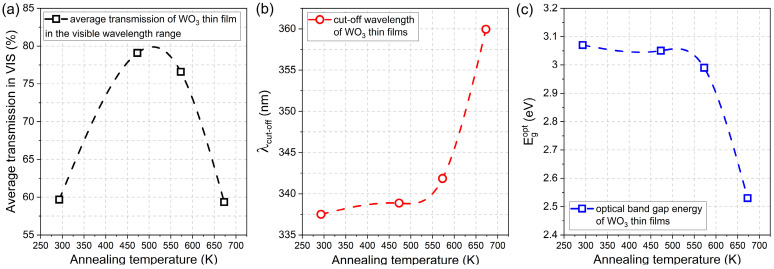
Comparison of (**a**) average transmission in the visible wavelength range, (**b**) fundamental absorption edge, and (**c**) optical band gap energies of WO_3_ thin films annealed at various temperatures.

The average transmission in the visible wavelength range, i.e., from 380 nm to 760 nm, was calculated for the as-prepared and annealed thin films. It was found that directly after deposition, the WO_3_ thin film had a transparency of ca. 60%, and it increased by almost 20% for samples annealed at 473 K and 573 K. However, further annealing at 673 K caused a decrease in average transmission to its initial value of approximately 60 % (Figure 5a). With an increase in the annealing temperature, the gradual shift of the position of the fundamental absorption edge was observed towards higher wavelengths causing a so-called red-shift from 337 nm to 360 nm (Figure 5b). Simultaneously, the inverse behaviour was observed for the optical band gap energy value as it decreased from 3.07 eV for the as-deposited thin film to 2.53 eV for WO_3_ annealed at 673 K (Figure 5c).

**Figure 6:**
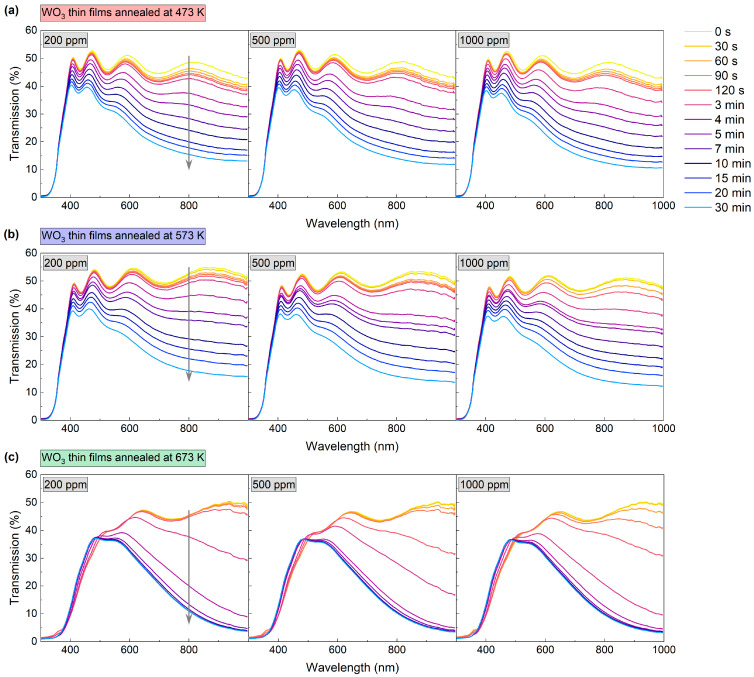
Transmission spectra during colouring of WO_3_ thin films annealed at (**a**) 473 K, (**b**) 573 K and (**c**) 673 K upon exposure to 200–1000 ppm H_2_. The grey arrow shows the direction of change in transmission in time during the colouring process.

The gas sensing performance of the WO_3_ thin films was characterised based on the measurement of transmittance spectra in the range of 300 nm to 1000 nm during the introduction of air and an H_2_/Ar mixture. H_2_ concentrations were: 200 ppm, 500 ppm, and 1000 ppm. The measurement was carried out in a cryostat at 423 K. The spectra were collected during the colouring of the coating at certain time intervals, as shown in Figure 6. Changes in transmission were most prominent at the beginning of the colouring cycle (up to about 10 min mark); therefore, in the initial stage, the spectra were collected more frequently. After the introduction of the H_2_/Ar gas mixture, a decrease in transmittance was observed for all of the analysed coatings. The greatest change was observed for wavelengths longer than 500 nm; for the coating annealed at 673 K, a slight increase in transmittance was observed for the wavelength range of 400 to 500 nm.

**Figure 7:**
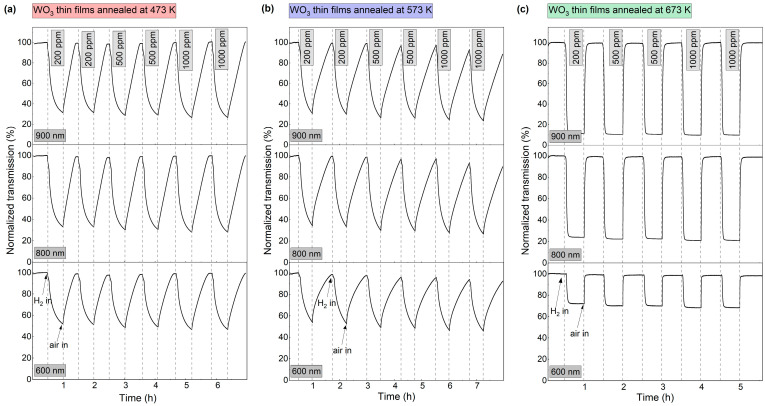
Variation in transmittance at 600 nm, 800 nm, and 900 nm during colouring/bleaching cycles of WO_3_ thin films annealed at (**a**) 473 K, (**b**) 573 K and (**c**) 673 K upon exposure to 200–1000 ppm H_2_.

To further characterise the dynamics of the gasochromic colouration process, the value of transmission coefficient at the wavelengths of 600 nm, 800 nm, and 900 nm was collected at 1 s intervals during several colouring/bleaching cycles (Figure 7). The duration of each colouring cycle was 30 min. The bleaching time was chosen independently for each sample to obtain a full recovery of the transmittance to the initial value. The highest decrease in transmittance for all thin films was observed at λ = 900 nm. For coatings annealed at 473 K and 573 K, the transmittance value at the examined wavelengths decreased during the entire colouring cycle. For the coating annealed at 673 K, a plateau was reached after ca. 7 min of exposure to hydrogen gas and the recovery of the baseline transmittance after air introduction was very fast (<5 min). The calculated sensing response was in the range of 3.2 to 3.9 for the coatings annealed at 473 K and 573 K and from 8.8 to 10.2 for the coating annealed at 673 K.

**Figure 8:**
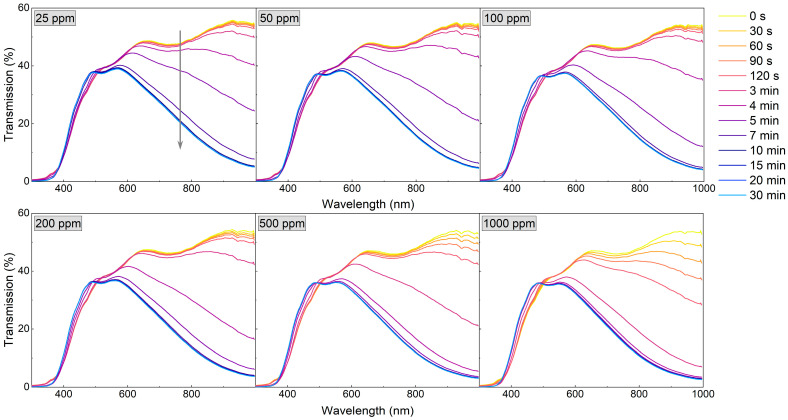
Transmission spectra during colouring of WO_3_ thin film annealed at 673 K upon exposure to H_2_ concentrations of 25 to 1000 ppm. The grey arrow shows the direction of change in transmission in time during the colouring process.

**Figure 9:**
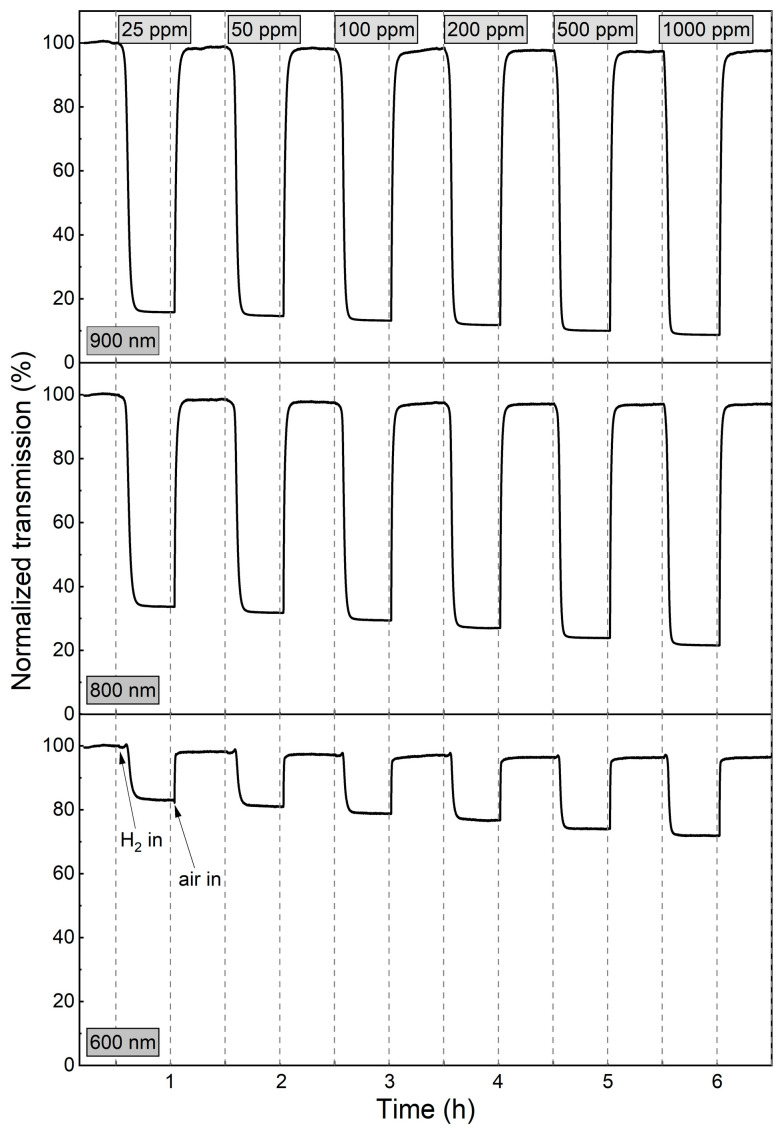
Variation in transmittance at 600 nm, 800 nm, and 900 nm during colouring/bleaching cycles of WO_3_ thin film annealed at 673 K upon exposure to H_2_ concentrations of 25 to 1000 ppm.

Since the WO_3_ coating annealed at 673 K was characterised by a very high sensor response and a fast response time to a hydrogen concentration as low as 200 ppm, the measurements were repeated, starting with even lower H_2_ concentrations (25–1000 ppm) (Figure 8 and Figure 9). For low H_2_ concentrations (25–100 ppm) during the first 4 min of exposure to H_2_, the observed transmission decrease was small and rather negligible. In the period between 4 and 11 min, there was a significant decrease in transmittance, and during the rest of the colouring cycle, the transmittance level was stable. For higher concentrations, the change of transmittance was observed earlier, and saturation was reached after approximately 9 min.

The sensor response, response time, and recovery time values, calculated for the wavelength of 900 nm, are shown in Table 1. The sensor response of the WO_3_ coatings annealed at 473 K and 573 K was in the range of 3.2 to 3.9. The response times for both films were in the order of 20 min. The sensor response of the WO_3_ thin films annealed at 673 K increased from 6.3 to 10.2 with increasing H_2_ concentration, while the response time decreased from 8.5 min to less than 4 min. We assume that the response times calculated for the lower H_2_ concentrations were longer due to the low partial hydrogen pressure achieved in the measurement chamber due to its large volume. This conclusion is confirmed by the dynamics of transmittance change for low and high H_2_ concentrations shown in Figure 9 and described above.

**Figure 10:**
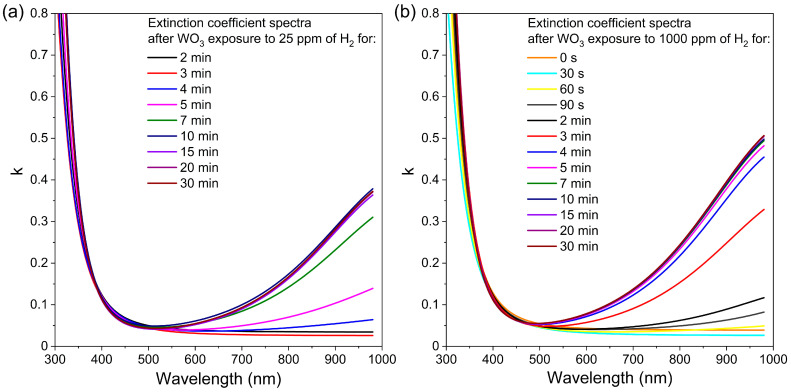
Variation in extinction coefficient in time during colouring of WO_3_ thin film annealed at 673 K upon exposure to H_2_ concentration of: (**a**) 25 ppm and (**b**) 1000 ppm.

In the case of transmission spectra, measurements performed during WO_3_ exposure to hydrogen showed a significant decrease in transmission for wavelengths greater than 500 nm. These results clearly show that during the colouration process, i.e., exposure to hydrogen, the absorption increases in time. For this reason, the extinction coefficient (*k*) was determined using a reverse engineering method employing SCOUT software ver. 4.17 (Wolfgang Theiss, Akwizgran, Germany). The changes in extinction coefficient spectra over time during colouring of the WO_3_ thin film annealed at 673 K upon exposure to 25 ppm and 1000 ppm of H_2_ are shown in Figure 10. This thin film has been chosen for further analysis because of its best sensor response, among others. In the case of WO_3_ thin film annealed at 673 K during the first 3 min of exposure to 25 ppm of H_2_, there is no sudden change of the extinction coefficient. However, after 4 min, the tail of the k started to increase significantly with increasing wavelength. The k value increased up to 10 min of exposure to H_2_, and then its value was stable to the end of the exposure cycle, meaning that absorption also increased up to some point and reached its plateau. For the thin films exposed to 1000 ppm of H_2_, the behaviour is very similar, with a major difference in the starting time of an increase in the k value, i.e., one minute instead of four after introducing hydrogen. In this case, the extinction coefficient increased after just 60 s of exposure to the H_2_ and reached a plateau at approximately 5 min. This, in turn, means that the response to hydrogen was obtained much faster than for the lower concentration. Such results are in very good correlation with the change of the transmittance shown in Figure 9 and the response time calculated for both concentrations.

Increased light absorption in optical materials can be caused by several factors, e.g., interband transition of electrical charge carriers, interaction with a crystal lattice, point defects in the structure, or absorption on free carriers of electrical charge. The release of free electrons often occurs during the reduction of the metal oxides, and it is a basis of the chromic effects, e.g., electrochromic or gasochromic. This, in turn, affects the light absorption in metal oxides such as tungsten oxide. In such a case, the Drude free-electron absorption model describing the interaction of the wave with the free-electron plasma can be employed to analyse the gasochromic effect. Based on the Drude model, after some transformations shown in [18] one can obtain the linear relationship of k on the wavelength, which can lead to calculations of the free charge carrier concentration in the thin films during exposure to hydrogen:

(1) $$k\approx \frac{e}{2\pi c}\sqrt{\frac{N}{\varepsilon_{o}m_{e}}}\lambda$$

where: e—elementary charge, c—speed of light, N—concentration of charge carriers, ε_0_—electrical permittivity of the vacuum, m_e_—effective electron mass, λ—wavelength.

**Figure 11:**
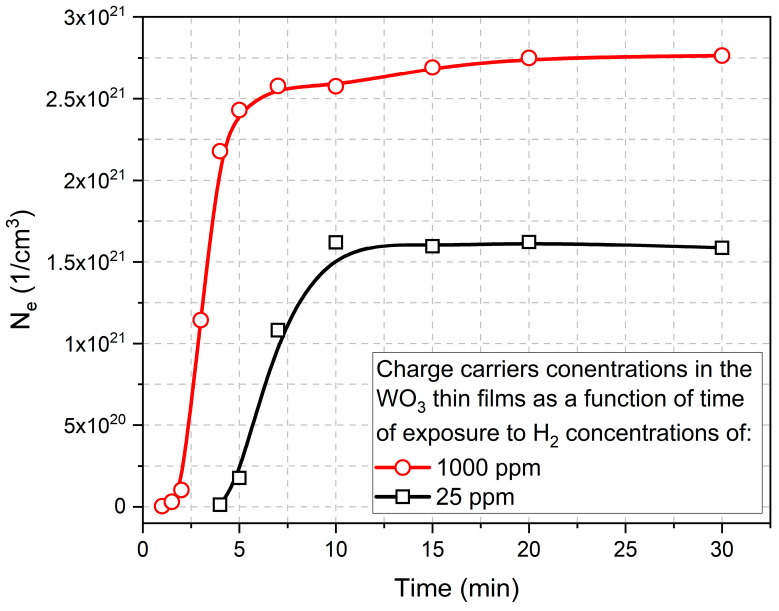
Electron concentration in the function of time of the colouring cycle of WO_3_ thin film annealed at 673 K calculated based on the results of the extinction coefficient.

Based on this equation, the slope of the characteristics of k vs. λ should be influenced by the value of the concentration of free carriers. Therefore, on the basis of the results presented in Figure 10, the electron concentration in the function of time of colouring cycle for WO_3_ thin films exposed to 25 ppm and 1000 ppm of H_2_ is shown in Figure 11. Those results are a good indication of the sensor response to the hydrogen, i.e., for higher concentrations of H_2_, the response time is much faster and much higher.

Georg, Graf, Wittwer, and others created the most cited model regarding the gasochromic phenomenon that occurs in WO_3_ under the influence of H_2_, which showed different stages of colouring and bleaching [29,30,31,32,33,34]. In principle, the gasochromic process relies on the chemical reactions of colouring and bleaching:

WO_3_ + 2H_2_ → WO_3_ + 4H → H_2_WO_3_ + 2H → WO_2_ + H_2_O  colouring (2)

2WO_2_ + O_2_ → 2WO_3_                   bleaching (3)

It is explained in several steps, which consist of the dissociation of the H_2_ molecule into two H atoms in the presence of a catalyst, ionisation of the H atoms, diffusion of H cations in the structure of the WO_3_ layer, and finally, formation of oxygen vacancies at the surface and movement of oxygen colour centres into the structure. As a result of these chemical reactions, the colouration of tungsten oxide occurs and tungsten ions are reduced from W^6+^ to W^5+^ or W^4+^. The recovery of the thin films was obtained in an atmosphere containing oxygen, which oxidised tungsten oxide to its initial stage.

**Figure 12:**
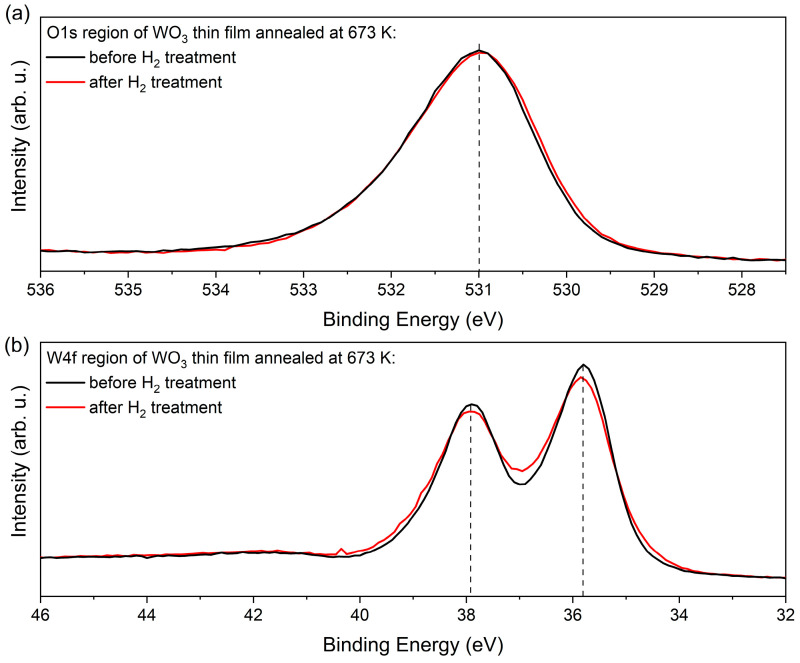
XPS measurements results of (**a**) O1s and (**b**) W4f regions of as-deposited and H_2_-treated WO_3_ thin films.

Figure 12 compares the XPS data of the W4f and O1s regions of the WO_3_ film annealed at 673 K before and after exposition to H_2_. Both spectra demonstrate that the electronic structure of the WO_3_ does not change a lot upon H treatment, as there is just a minor increase in the line width of about 200 meV. Remarkable is the fact that the data in Figure 12 exhibit a clear asymmetric line shape that appears on the respective high binding energy (BE) side of the core levels. In the W4f data, this asymmetric shape cannot be explained by contributions of (extremely unrealistic) higher oxidation states than W^6+^. These findings and, in particular, the absence of pronounced contributions of W^4+^ and W^5+^ point to a different explanation of the H-induced colouring and gasochromic mechanisms than proposed in [29,30,31,32,33,34].

Instead, the asymmetric line profile in the core level data point to the existence of quasi-metallic contributions in the electronic structure of WO_3_ that causes the screening on the high BE side of the core levels [35]. Such screening mechanisms and the asymmetric line shapes affect the W4f of the O1s signal in a similar way, as evident in Figure 12.

The asymmetric screening is not expected in the pure ionic WO_3_ material. Its evidence is a clear signature for the existence of extended defect states that allow a charge distribution over several atomic sites. In WO_3_, such intrinsic defect states (IDS) are located within the ionic gap below the ionic conduction band minimum (CBM). They form the states beyond the optical gap, and their onset defines the optical band gap [36,37]. The IDS states are cross-linked by covalent bonds and accommodate delocalised charge carriers. This enables an independent identification by their quasi-continuous density of states within the ionic band gap in the resonant photoemission spectroscopy (resPES) data.

For WO_3_ presence of IDS is proposed in a theoretical study presented in [38]. For O-rich and O-poor environments, they appear either localised or delocalised, respectively. A similar mechanism of charge donation via intrinsic defect states has recently been proposed to explain the chemical interaction of Al_2_O_3_ with organic-inorganic methylammonium lead trihalide perovskite films [39].

With respect to the H-sensing mechanism in WO_3_, the charge donation of the H atom then proceeds into/from these intrinsic defects. The colouration effect discussed here takes place in the diluted hydrogen atmosphere, and this is in line with the absence of structural/chemical change of the tungsten oxide. The sensing data and the observed increase/changes in the optical parameters are directly related to the changes/increase in the free charge carriers concentration in the IDS band.

The density of the IDS and their delocalised and covalent charges is represented by the ionicity factor f_i_ [40]. We can apply a recipe to determine f_i_ from the O1s core level data that is introduced in Ref. [41] that correlates the carrier densities in the covalent and ionic subsystems by correlating the contribution at higher BE to that of the main peak. The f_i_ parameter was calculated following the method given by Schmeisser et al. [41] and analysing the O1s spectrum. This method concerns the two contributions in the O1s core levels (O^∗^ = I_ionic_/(I_cov_ + I_ionic_)) [41], which are typical for many oxide systems. Generally, ionic systems appear at lower binding energies, while covalent systems exhibit larger binding energies. For the data shown in Figure 12, we obtained an ionicity factor of f_i_ = 0.78. with its covalent part (1 − f_i_) = 0.22. This rather large number of covalent carriers on WO_3_ causes the quasi-metallic screening of the WO_3_ core levels. The resulting asymmetric broadening is best observable on single O1s signals and on the very narrow line widths of the W4f signals. A similar analysis of the O1s core levels in a series of different oxide systems has recently found f_i_ values between 0.5 and 0.9, and this demonstrates the general applicability of the concept of covalent bonded carriers occupying IDS defect bands within the underlying basic ionic system [41].

In order to further analyse the cross-link between the optical absorption/transmission data and the population of the IDS in terms of hydrogen sensing and gasochromic properties, we plan to use synchrotron-based (improved energy resolution) resPES investigation of WO_3_ films (work in progress). From this single method, the carrier densities of the covalent IDS sites can be related to those determined here from the extinction data (Figure 11).

A comparison of the presented results from previously published works is shown in Table 2. It can be seen that, while the response and recovery times obtained in this work are longer than some of those reported previously, e.g., by Nishizawa et al. [15,42] and Wisitsoorat et al. [11], they were obtained for hydrogen concentrations as low as 25 ppm, which is 40–400 times lower than those usually used (0.1–1%). The gasochromic performance is similar to the results shown by Cho et al. [6] (ΔT = 57%), Okazaki et al. [13] (ΔT = 60%), Yamaguchi et al. [12] (T_H2_/T_air_ = 0.1) and Takano et al. [43] (T_H2_/T_air_ = 0.1). However, the H_2_ concentrations used in those papers were also 40–1600 times higher than those used in our work.

## 4. Conclusions

WO_3_ thin films were deposited using gas impulse magnetron sputtering, which allowed for the preparation of amorphous, smooth, and homogeneous thin films. Post-process annealing at 573 K caused a start of the phase transition from amorphous to nanocrystalline WO_3_ with a crystallite size of approx. 21 nm, a considerable increase in the average transparency in the visible wavelength range related to the oxidation of the thin films, and a slight decrease in the optical band gap energy. In turn, annealing at 673 K resulted in a rather fully crystalline structure with a slightly increased crystallite size of 23 nm, a change in the cross-section morphology from amorphous to columnar while maintaining a featureless and homogenous surface, and a significant decrease in the average transparency and optical band gap energy.

Investigations of gasochromic properties performed for WO_3_ thin films covered with a Pd catalyst overlayer showed that annealing at 473 K and 573 K did not change the optical response of thin films in a major way. In both cases, the SR values were very similar, whereas, for higher temperatures, an improvement in sensor response and recovery times was observed. Beyond that, based on the calculated extinction coefficient for WO_3_ at various times of gasochromic process, it was possible to determine the change of the concentration of the free charge carriers concentration and that it increases with an increase in the time of the H_2_ poisoning cycle.

Next, an increase in the annealing temperature to 673 K resulted in obtaining the best optical gas sensor toward hydrogen with the SR of 6.3 and 10.2 for 25 and 1000 ppm of H_2_ with response and recovery times of 3.5 and 2.5 min, respectively. A very high SR of 6.3 for H_2_ concentrations as low as 25 ppm is the lowest value found by us in the literature for WO_3_ optical gas sensor based on the gasochromic effect. Such an increase in the performance of H_2_ gas sensing properties for the annealed at 673 K WO_3_ film could be attributed to the creation of covalently bonded intrinsic defect states in the WO_3_ film during the transition from amorphous to the crystalline phase. Indeed, in the XPS results collected on the annealed at 673 K WO_3_ film, we have found about 22% of the covalently bonded intrinsic defect states in the ionic matrix of the WO_3_ film. This work is in progress.

## Figures and Tables

**Table 1:** Summary of gas sensor parameters of WO_3_ thin films annealed at various temperatures, calculated for λ = 900 nm.

Annealing Temperature (K)	H_2_ Concentration (ppm)	SR	t_response_ (min)	t_recovery_ (min)
473	200	3.2	17.5	21.5
	500	3.4	18.5	26
	1000	3.8	19	31
573	200	3.3	21	36
	500	3.7	20	40
	1000	3.9	20	40.5
673	25	6.3	8.5	5
	50	6.8	7.5	4.5
	100	7.4	6	3.5
	200	8.8	4.5	3.5
	500	9.5	4	3
	1000	10.2	3.5	2.5

**Table 2:** Gasochromic performance of WO_3_ thin films.

Material	Deposition Method (WO_3_)	H_2_ Concentration	Operating Temperature	Gasochromic Performance	Response Time	Recovery Time	Ref.
Au/WO_3_	RF magnetron sputtering	0.6–1%	200 °C	Δ Cumulative absorbance = 2.5% (0.06% H_2_)	120 s (1% H_2_)	300 s	[14]
Pt/WO_3_	Low-temperature chemical fabrication	4%	RT	ΔT = 70%	5 s	5 s	[42]
	RF magnetron sputtering	0.1–1%	100 °C	Δ Cumulative absorbance = 51% (0.1% H_2_)	60 s (1% H_2_)	90 s (1% H_2_)	[11]
	Sol-gel	4%	RT	ΔT = 60%	10 s	>150 s	[13]
	Sol-gel	1%	RT	T_H2_/T_air_ ≈ 0.1	60 s	>150 s	[12]
	RF magnetron sputtering	0.06–1%	100 °C	Δ Cumulative absorbance = 1.5% (1% H_2_)	–	–	[44]
Pd/WO_3_	Electron beam evaporation	1%	RT	ΔT = 57%	400 s	16 s	[6]
	Low-temperature chemical fabrication	4%	RT	ΔT = 70%	<60 s	5 s	[15]
	RF magnetron sputtering	0.1–1%	RT	T_H2_/T_air_ = 0.1 (1% H_2_)	<10 s	–	[43]
	RF magnetron sputtering	0.06–1%	100 °C	Δ Cumulative absorbance = 20% (1% H_2_)	–	–	[44]
	Gas impulse magnetron sputtering	0.0025%	150 °C	ΔT = 45%, T_H2_/T_air_ = 0.16, Δ Cumulative absorbance = 12.6%	510 s	300 s	This work
	Gas impulse magnetron sputtering	0.1%	150 °C	ΔT = 48%, T_H2_/T_air_ = 0.09, Δ Cumulative absorbance = 13.8%	210 s	150 s	This work

## Data Availability

The data presented in this study are available on request from the corresponding author.
